# A novel multipoint mucosal bridge technique in endoscopic submucosal tunneling dissection for esophageal lesions

**DOI:** 10.1055/a-2607-8461

**Published:** 2025-06-13

**Authors:** Xiangqiang Liu, Dongtao Shi, Xiaoqiang Yang, Rui Li, Hongwu Zhu

**Affiliations:** 1 Department of Gastroenterology, General Hospital of Southern Theater Command, PLA, Guangzhou, China; 2 Department of Gastroenterology, The First Affiliated Hospital of Soochow University, Suzhou, China

A 67-year-old man with a 7-month history of acid reflux and belching underwent esophagogastroduodenoscopy, revealing two mucosal lesions at 32–38 cm from the incisors (2.0 × 1.5 cm, 4.0 × 2.0 cm; Fig. 1 ). Biopsy confirmed severe squamous epithelial dysplasia with focal carcinoma. We employed an innovative modification of the endoscopic submucosal tunnel dissection (ESTD) technique 1 , which we have termed “multipoint mucosal bridge method” ESTD (MPMB-ESTD), to achieve en bloc resection of the lesions ( Video 1 ).

**Fig. 1:**
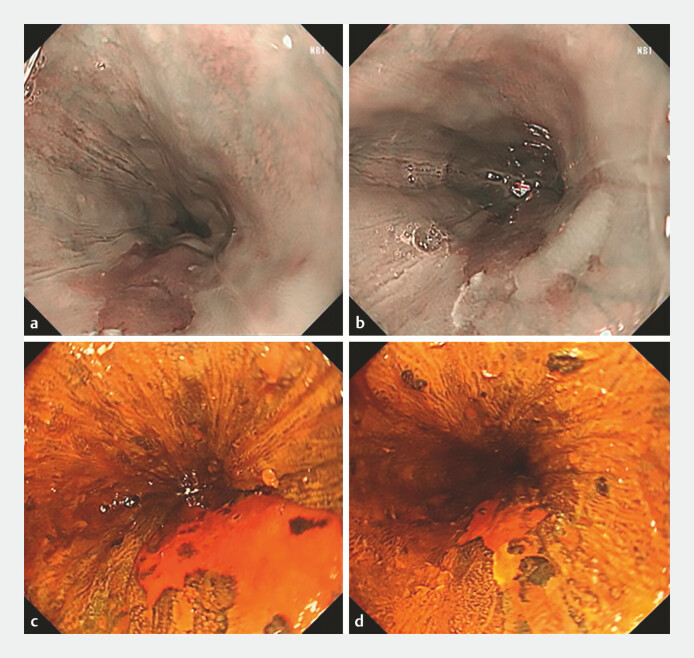
Esophageal mucosal lesions measuring 2.0 × 1.5 cm and 4.0 × 2.0 cm were identified at 32–38 cm from the incisors. **a, b** Narrow-band imaging showing the lesions. **c, d** Corresponding iodine-stained images highlighting the lesion margins.

Endoscopic en bloc resection of esophageal lesions using the multipoint mucosal bridge method of endoscopic submucosal tunnel dissection, demonstrating partial circumferential incision with preserved mucosal bridges, submucosal tunneling dissection, and lesion stabilization.Video 1

The MPMB-ESTD technique was performed as follows. 1) Marking lesion margins: lesion boundaries were marked approximately 0.5–1 cm beyond the visible margins. 2) Partial circumferential incision: following submucosal injection, a near-complete circumferential incision was made, preserving four mucosal “bridges” at the lesion corners to serve as stabilizing anchors. 3) Tunnel creation and submucosal dissection: a proximal incision was used to establish a submucosal tunnel. Dissection was performed within the tunnel using repeated injections and gradual peeling of the submucosal layer, ensuring precision and maintaining lesion stability. 4) Final resection: the mucosal bridges were severed effortlessly after submucosal dissection, completing en bloc resection of the lesion ( Fig. 2 ).

**Fig. 2:**
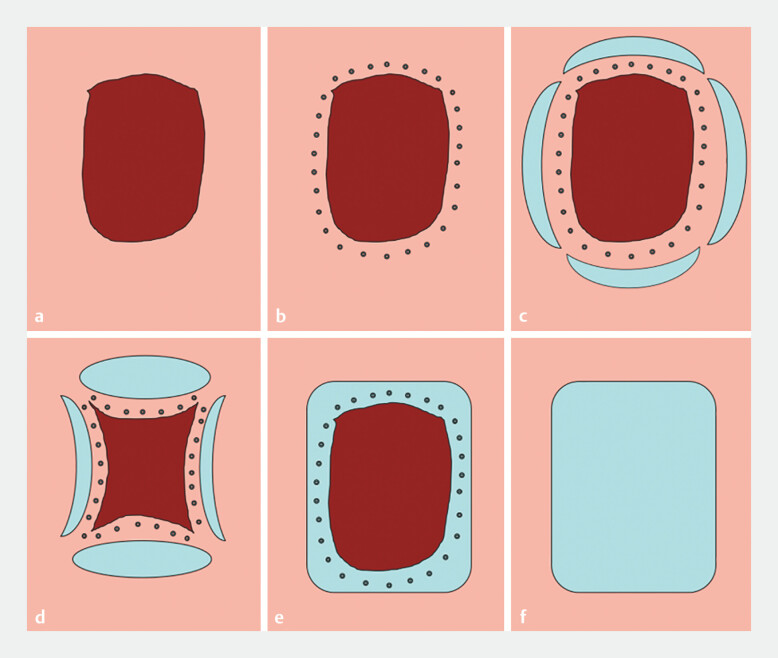
Schematic diagram of the operational steps of the multipoint mucosal bridge method of endoscopic submucosal tunnel dissection. **a** Clear exposure and observation of the lesion. **b** Marking the margins of the lesion. **c** After submucosal injection, performing a near-circumferential incision around the lesion while preserving four “mucosal bridges” at the corners of the lesion. **d** Establishing a submucosal tunnel using the proximal incision as the entry point and completing dissection of the lesion within the tunnel. **e** Achieving complete en bloc resection of the lesion by easily severing the mucosal bridges. **f** Postoperative management of the wound surface.

Postoperative pathology confirmed a 6.0 × 3.5 cm lesion with poorly differentiated squamous cell carcinoma infiltrating the muscularis mucosae to a depth of 0.6 mm. All lateral and basal margins were free of carcinoma.

The MPMB-ESTD technique offers a significant improvement over conventional ESTD by addressing key limitations while maintaining the benefits 2 3 . By preserving the lateral mucosa and submucosa, the technique minimizes unnecessary tissue damage, enhances precision, and promotes better postoperative healing. The mucosal bridges provide structural support, simplifying resection, improving safety, and reducing procedural complexity. Additionally, the technique requires no extra equipment, making it cost effective. This novel approach enhances efficiency and operability, and demonstrates potential advantages in the en bloc resection of esophageal lesions.

Endoscopy_UCTN_Code_TTT_1AO_2AG_3AZ

## Notes

Endoscopy E-Videos https://eref.thieme.de/e-videos
*E-Videos* is an open access online section of the journal *Endoscopy* , reporting on interesting cases and new techniques in gastroenterological endoscopy. All papers include a high-quality video and are published with a Creative Commons CC-BY license. Endoscopy E-Videos qualify for HINARI discounts and waivers and eligibility is automatically checked during the submission process. We grant 100% waivers to articles whose corresponding authors are based in Group A countries and 50% waivers to those who are based in Group B countries as classified by Research4Life (see: https://www.research4life.org/access/eligibility/ ). This section has its own submission website at https://mc.manuscriptcentral.com/e-videos .

## References

[1] Zhai YQ Li HK Linghu EQ Endoscopic submucosal tunnel dissection for large superficial esophageal squamous cell neoplasms World J Gastroenterol 2016 22 435 445 10.3748/wjg.v22.i1.435 26755889 PMC4698506

[2] Wang J Zhu XN Zhu LL Efficacy and safety of endoscopic submucosal tunnel dissection for superficial esophageal squamous cell carcinoma and precancerous lesions World J Gastroenterol 2018 24 2878 2885 10.3748/wjg.v24.i26.2878 30018482 PMC6048426

[3] Pioche M Mais L Guillaud O Endoscopic submucosal tunnel dissection for large esophageal neoplastic lesions Endoscopy 2013 45 1032 1034 10.1055/s-0033-1344855 24165887

